# eHealth for Breast Cancer Survivors: Use, Feasibility and Impact of an Interactive Portal

**DOI:** 10.2196/cancer.5456

**Published:** 2016-05-10

**Authors:** Wilma Kuijpers, Wim G Groen, Hester SA Oldenburg, Michel WJM Wouters, Neil K Aaronson, Wim H van Harten

**Affiliations:** ^1^ The Netherlands Cancer Institute Division of Psychosocial Research and Epidemiology Amsterdam Netherlands; ^2^ The Netherlands Cancer Institute Division of Surgical Oncology Amsterdam Netherlands; ^3^ University of Twente Department of Health Technology and Services Research Enschede Netherlands

**Keywords:** eHealth, interactive portal, feasibility, breast cancer survivor

## Abstract

**Background:**

MijnAVL is an interactive portal including patient education, overview of appointments, access to the electronic medical records (EMR), patient-reported outcomes, plus feedback and physical activity support.

**Objective:**

With this study we aimed to evaluate the use, feasibility, and impact of MijnAVL among breast cancer survivors.

**Methods:**

We included survivors currently or recently treated with curative intent, who completed questions on sociodemographics, patient activation (PAM), quality of life (SF-36), and physical activity (IPAQ). MijnAVL could be used noncommittally for four months. Log data were collected retrospectively and participants completed questions on acceptability, satisfaction, and the PAM, SF-36 and IPAQ.

**Results:**

Ninety-two women (mean age 49.5 years, 59% on-treatment) participated, with a mean number of logins of 8.7. Overview of appointments (80% of participants) and access to the EMR (90%) were most frequently used and most highly valued. Average website user satisfaction was 3.8 on a 5-point scale. Although participants reported having more knowledge and experiencing more control of their situation after using MijnAVL, PAM scores did not change significantly. Three domains of the SF-36 (role functioning - emotional, mental health, and social functioning) and median vigorous physical activity improved significantly over time. The burden of MijnAVL for professionals was limited.

**Conclusions:**

User experiences were positive and exposure to MijnAVL was accompanied by improvements in three quality of life domains and vigorous physical activity. Tailored features may be needed to enhance the usefulness and efficacy of MijnAVL. Research with a controlled design is needed to confirm our findings.

## Introduction

Cancer survivors are individuals who are undergoing, or have completed, the primary treatment phase [1] and they are often considered to have a chronic disease. Survivors may suffer from acute (eg, nausea, hair loss, pain), long-term (eg, fatigue, anxiety, sexual problems) and late effects (eg, second malignancies, cardiovascular disease) caused by cancer or its treatment. These effects often have an impact on health status and quality of life, and might even require professional help [2]. Current models of health care, with a focus on detection and treatment of acute disease, seem no longer sustainable given the increasing number of people with a chronic disease like cancer [3]. Additionally, the time of health professionals is limited and the costs involved in professional help are rising.

A transition to patient-centered models of care in which cancer survivors play a more active role in their care process is needed. This idea could be referred to as *patient empowerment*, which implies that cancer survivors’ autonomy is respected by health professionals and that survivors have the knowledge and the psychosocial and behavioral skills needed to positively influence their health status [4]. Research has shown that interventions aimed at improving patient empowerment can have a positive effect on health behavior and health outcomes [5].

To support patient empowerment, it may be helpful to utilize information and communication technologies in health care (eHealth) [6,7]. An important advantage of eHealth is that tailored (sometimes also referred to as personalized) information and interventions can be more easily provided. In the Netherlands, approximately 95% of the population has access to the Internet. Although its use is highest among younger age groups, 75% of individuals aged 65-75 also regularly use the Internet [8]. Considering the mean age at breast cancer diagnosis in the Netherlands is 61 years, and many breast cancer survivors already use the Internet to find cancer-related information [9], they are an appropriate target population for eHealth services. To date, eHealth applications for breast cancer survivors have focused primarily on online (peer) support [10], patient education [11], or singular aspects of empowerment, such as psychological adjustment [12].

In the Netherlands Cancer Institute (Antoni van Leeuwenhoek hospital; AVL) we have developed an eHealth application to support cancer survivors during the whole cancer trajectory, known as MijnAVL (MyAVL in English). MijnAVL is a secured portal that provides survivors with personalized information, insight into their health status, and tailored physical activity advice. The aim of this study was to evaluate the use, feasibility, and impact of MijnAVL among breast cancer survivors.

## Methods

### Participants and Procedures

We invited women with histologically confirmed breast cancer who were currently receiving curative treatment (surgery, radiotherapy and/or chemotherapy) or had received such treatment 3-12 months ago. Survivors received a letter in which the purpose of the study was explained, followed by a phone call from the researchers to discuss participation and check further eligibility criteria (ie, having a computer and Internet, mastery of the Dutch language). Women with cognitive disorders or emotional instability were excluded. Those declining participation were asked to indicate their main reason for non-participation. The Institutional Review Board approved the study and participants provided written informed consent.

We used a pretest-posttest design. Upon completion of the baseline questionnaire, participants could access MijnAVL noncommittally for 4 months, after which they were asked to complete a post-test questionnaire. We organized a focus group with a random selection of participants to obtain more detailed feedback about MijnAVL. Health professionals (medical oncologists, surgeons, radiotherapists and nurse specialists) were asked to complete a questionnaire about the impact of MijnAVL on their work.

### Intervention

**Figure 1:**
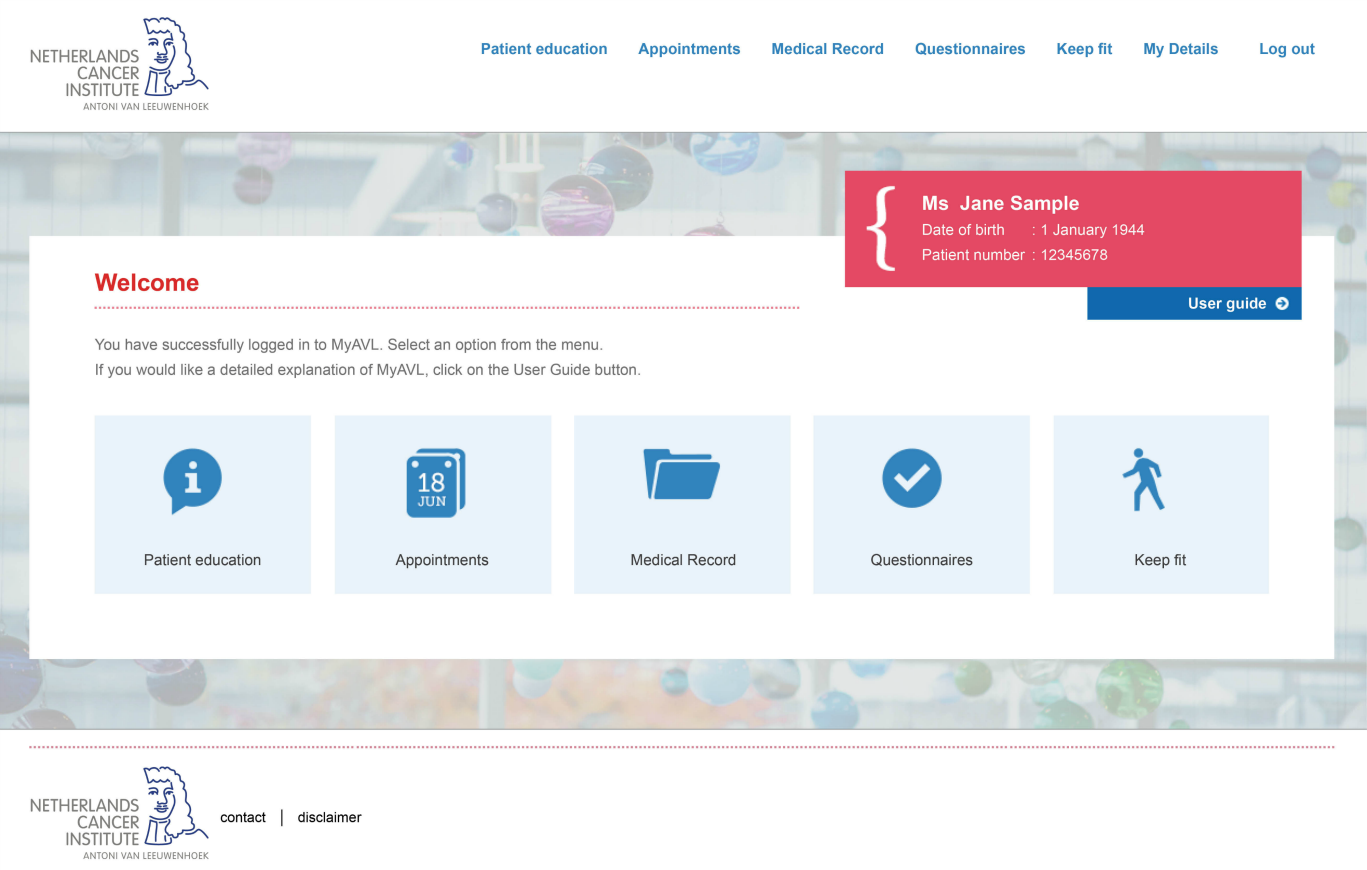
Screenshot of the homepage of MijnAVL.

The development, content, and layout of MijnAVL have been described in detail previously [13,14]. The system includes personalized educational material (eg, about their disease, their treatment, and possible side effects) and an overview of past and upcoming appointments. Users can also access parts of their electronic medical record (EMR) including radiology, pathology and lab results, conclusions from multidisciplinary meetings, and a medication overview. This information is supported by a dictionary and placed on MijnAVL with a delay of two weeks to make sure that there is sufficient time to first discuss the results with a health professional. Additionally, users receive a request by email to complete patient-reported outcomes (PROs) about their quality of life at regular intervals. In this email, it is pointed out that these PROs can yield useful information about their health status. If participants have not completed the PROs within one week, they receive an email reminder. Both participants and health professionals are provided with a summary of the PRO scores and are encouraged to discuss these results. The physical activity support program automatically provides tailored advice based on a set of questionnaires assessing clinical characteristics, nutritional status, physical activity levels, and motivation. The advice is aimed at influencing constructs from Social Cognitive Theory - behavioral, environmental, and personal factors [15] and the Theory of Planned Behavior - attitude, subjective norm, and perceived behavioral control [16]. For example, an individual preparing to become physically active is encouraged to think of possibilities for being physically active in her home setting, whereas another individual who is already physically active and needs to maintain her level of activity is advised to (continue to) use goal setting in order to stay motivated. Figure 1 shows a screenshot of the homepage of MijnAVL.

### Assessments

The use of MijnAVL (number and duration of logins, pages visited, and questionnaires completed) was automatically logged for each participant and clinical information (Union for International Cancer Control stage, type of treatment, and time since treatment) was obtained from the EMR.

#### Baseline Questionnaire

First, sociodemographics such as marital status, education level, and employment status were obtained. Internet use was measured in terms of frequency and duration. The Dutch version of the eHealth Literacy Scale (eHEALS) was used to assess users’ ability to find and evaluate online health information. The eHEALS score ranges between 0 and 40; 40 being the highest score [17]. We assessed expectations about MijnAVL with questions covering a range of issues derived from the Unified Theory of the Acceptance and Use of Technology (UTAUT) framework. This framework includes factors that directly or indirectly predict the behavioral intention to use a technology and/or the actual use of that technology [18]. The following factors were assessed: performance expectancy or usefulness (eg, “MijnAVL will be a valuable supplement”), effort expectancy (ease of use, eg, “MijnAVL will be easy to use”), social influence (eg, “People who are important to me will encourage me to use MijnAVL”), self-efficacy (eg, “I have the ability to use MijnAVL”), attitude (eg, “It will be a good idea to use MijnAVL”), and intention (eg, “I intend to use MijnAVL as often as needed”). Facilitating conditions were not measured, as the availability of a computer and Internet access were inclusion criteria for the study. Response options ranged from 1 (completely disagree) to 7 (completely agree).

Data on the impact of MijnAVL was examined with three validated questionnaires. The patient activation measure (PAM) was used to measure patient empowerment in terms of knowledge, skills, and confidence in self-management [19]. The PAM consists of 13 questions with response options varying from 1 (strongly disagree) to 4 (strongly agree). Responses are converted to a total score ranging from 0 to 100, with higher scores representing more activation.

Quality of life was assessed with the Short-Form 36-Item Health Survey (SF-36), which consists of eight scales (eg, physical functioning, mental health, and vitality) that are scored from 0 to 100, with higher scores being more favorable [20].

Physical activity was assessed with the International Physical Activity Questionnaire (IPAQ), which measures the frequency (days per week) and duration (minutes) of physical activity during the last seven days in the following domains: work, transportation, work at home, and leisure activities [21]. Different levels of exercise (walking, moderate, vigorous, and total) were calculated and expressed in Metabolic Equivalent of Task (MET)-minutes per week (a product of exercise intensity and duration).

#### Post-Intervention Questionnaire

Participants reported on their use (eg, frequency, duration, features used) of MijnAVL. Satisfaction with MijnAVL was measured with the website user satisfaction questionnaire (WUS), assessing 11 dimensions of satisfaction such as information comprehensibility, ease of use, and web site structure [22]. Experiences were assessed with questions based on the UTAUT framework that were an adapted version of the ones that were used to measure expectations at baseline (ie, rewritten in the past tense). We also posed specific questions on acceptability of each feature, for example, about the quality, timing, and comprehensibility of information, and about the usefulness of the feedback on PROs and IPAQ. These questions could be answered on a 5-point scale ranging from 1 (completely disagree) to 5 (completely agree). Participants rated the different features of MijnAVL and finally completed the PAM, the SF-36, and the IPAQ.

#### Focus Group

We discussed experiences with MijnAVL more thoroughly and gathered information on possible improvements and facilitators of long-term use. The focus groups were audio-taped and notes were taken.

#### Questionnaire for Health Professionals

Involved professionals received a short questionnaire about the impact of survivors’ access to MijnAVL on their work (eg, “Did you receive questions about MijnAVL and its content?” and, “Did your workload increase?”).

### Statistical Analyses

We used descriptive statistics to characterize the study sample in terms of clinical and sociodemographic variables, and to report expectations, satisfaction, experiences, use, and measures of preliminary efficacy. Given the non-normal distribution of the data, IPAQ results are reported as median MET-minutes per week. We used the Chi-square statistic and Student’s t-tests to examine possible differences between the groups on-treatment versus off-treatment in clinical and sociodemographic characteristics at baseline, as well as in expectations and experiences. We assessed changes over time on the PAM and the SF-36 with paired-samples t-tests, and on the IPAQ with the Wilcoxon Signed Rank test. These tests were also used to perform subgroup analyses (on-treatment vs off-treatment). We also checked, on an individual level, whether physical activity levels increased or decreased. We considered P-values <0.05 to be statistically significant and used the Statistical Package for the Social Sciences version 22.

Notes from the focus groups were combined with the qualitative comments from the questionnaires to highlight the most important issues. Health professionals’ questionnaire responses were summarized using descriptive statistics and a qualitative analysis of answers to the open-ended questions.

## Results

### Participants

**Figure 2:**
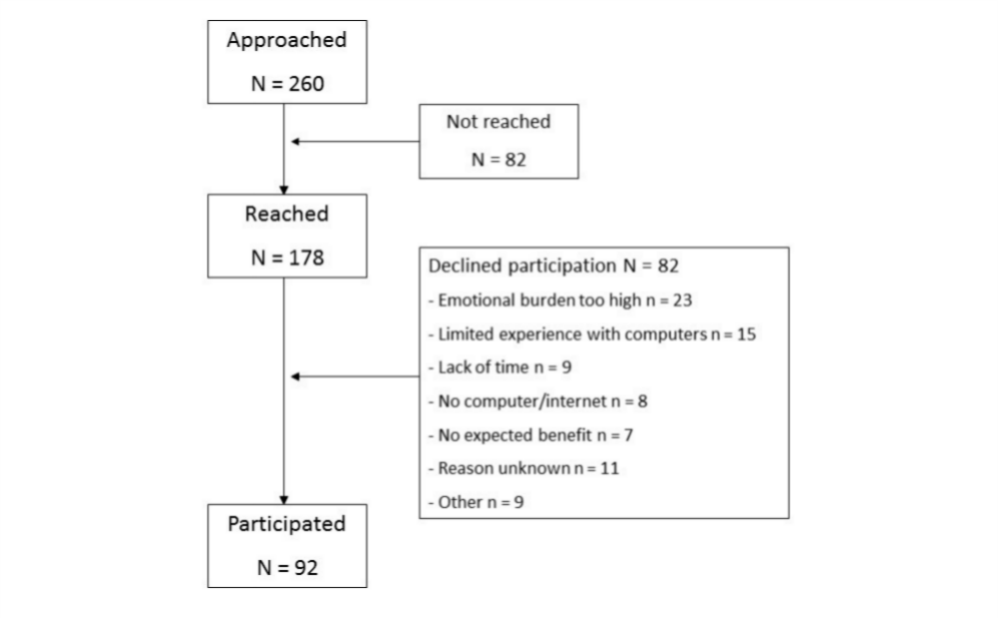
Participant flow chart.

Between January 2014 and April 2015, 260 women were invited to participate, of whom 92 agreed (response rate 35.4%). Figure 2 displays the flow of patient recruitment and participation.

Fifty-nine percent of the participants were on-treatment, and those off-treatment had completed primary treatment, on average 6.2 (SD 3.0) months earlier. The sociodemographic and clinical characteristics of the sample are reported in Table 1.

**Table 1:** Baseline characteristics (N=92).

Characteristic	%
Marital status	
Relationship, living together	64.1
Relationship, not living together	8.7
Single	19.6
Divorced	5.4
Widow	2.2
	
Education	
Compulsory or less	6.5
Post compulsory	20.7
University or college	72.8
	
Employment status=	
Full-time job	28.2
Part-time job	40.0
Homemaker	4.7
Retired	10.6
Unemployed	4.7
Voluntary work	1.2
Disabled	10.6
	
Union for International Cancer Control stage	
Ductal Carcinoma In Situ (DCIS)	11.0
I	28.6
II	45.1
III	15.4
	
Type of treatment	
Surgery	19.1
Surgery + Radiotherapy	21.3
Surgery + Chemotherapy	15.7
Surgery + Chemotherapy + Radiotherapy	43.8
Hormonal therapy	56.2
Immunotherapy	7.9

Participants’ mean age was 49.5 (SD 11.4) years, and the majority were highly educated and had a job. All participants had undergone surgery, and 80% (74/92) had adjuvant treatment. Ninety-six percent (88/92) had used the Internet for more than 3 years, 86% (79/92) were using it daily, and mean eHealth literacy was 30.8 (SD 4.4). Sociodemographic characteristics did not differ significantly between those on-treatment and off-treatment. The post-intervention questionnaire was completed by 87% (80/92) of participants.

### Expectations and Experiences with MijnAVL

Participants’ expectations and experiences, based on UTAUT, are shown in Table 2. Expectations were generally high, except for social norm. Experiences regarding social norm and intention were significantly lower than the expectations. For the remaining components, experiences were comparable to expectations.

**Table 2:** Expectations of and experiences with MijnAVL.

UTAUT component	Expectation	Experience	
	Mean (SD)	Mean (SD)	*P-value*
Ease of use	5.89 (0.91)	5.71 (1.17)	.228
Usefulness	5.13 (0.95)	4.86 (1.34)	.097
Attitude	6.10 (0.89)	5.89 (1.13)	.087
Social norm	4.16 (1.39)	2.82 (1.50)	.000
Self-efficacy	6.29 (0.80)	6.36 (0.81)	.524
Intention	6.43 (0.70)	5.55 (1.62)	.000

### Use

The majority of participants (69/92, 75%) indicated that it was easy or very easy to log on to MijnAVL, and 90% (83/92) used MijnAVL without any assistance. Statistics regarding use are shown in Table 3. Use varied widely across participants; the number of logins ranged from 0-62 and the duration of use from 2-38 minutes. Participants on-treatment used MijnAVL and its features more often than those off-treatment (except for accessing quality of life scores), however the visits of those off-treatment lasted longer. PRO completion rates were high and similar for those on-treatment and off-treatment (77% and 83%, respectively). The overview of appointments and the EMR were accessed most frequently and used by the largest number of participants (80% and 90%, respectively).

**Table 3:** Use of MijnAVL

	On-treatment (N=46)	Off-treatment (N=37)	Rating (N=92)
	Mean (SD)	Mean (SD)	1-10 scale
Number of logins (in 4 months)	10.9 (12.7) ^a^	5.6 (3.7)^b,c^	Not applicable
Mean duration of login (minutes)	11.3 (6.5)	15.2 (8.9)^d^	Not applicable
Patient education	4.0 (6.3)^e^	1.8 (1.3)^d,e^	6.9
Overview of appointments	8.8 (11.2)^e^	3.3 (2.1)^c,e^	8.7
Access to EMR	8.7 (11.3)^e^	3.8 (3.0)^c,e^	7.9
Quality of life scores	3.5 (3.9)^e^	2.4 (2.3)^e^	6.8
Physical activity support program	4.4 (3.7)^e^	2.4 (1.8)^c,e^	6.1

^a^N=54

^b^N=38

^c^Different from on-treatment; *P*<.001

^d^Different from on-treatment; *P*<.05

^e^These numbers reflect how many times the specific features were used during the 4-month study period.

### Satisfaction

The overall mean score for the WUS was 3.8 (SD 0.44) on a 5-point scale. The ease of use, website structure, and accuracy domains were particularly highly valued. MijnAVL as a whole was rated 7.6 on a 10-point scale, with the overview of appointments and access to the EMR being rated highest. Acceptability of MijnAVL was good in terms of perceived usefulness and comprehensibility, although the graphical presentation of PRO results was less well understood, and the information from the EMR raised questions for approximately 40% of participants. The EMR information and educational materials did not lead to anxiety for the majority of participants, and most participants indicated that access to this information increased their knowledge about, and control over, their disease. Participants reported that the patient education and physical activity advice could be improved by adapting it more to one’s personal situation and by making the advice more interesting, concise, and motivating. Participants expressed appreciation for the opportunity to complete PROs at home, but some indicated disappointment that their health professional(s) did not discuss the results with them.

### Impact

An overview of the outcome measures at baseline and post-intervention are presented for the total group (Table 4) and for participants on-treatment and off-treatment separately (Table 5). Scores on the PAM did not change significantly over time. Three domains of the SF-36 improved significantly over time: role functioning–emotional (*P*=.021) and mental health (*P*=.000) improved for those during treatment, and social functioning for those after treatment (*P*=.001). Median vigorous physical activity increased significantly from 0.0 to 360.0 MET-minutes per week for the total group (*P*=.017), although this effect was not apparent in the subgroup analyses. However, the total amount of physical activity actually decreased over time for about half of those on-treatment, and for about one-third of those off-treatment.

**Table 4:** Outcome measures for the total group.

	Total group (N=73)	
	Baseline	Post-intervention
	Mean (SD)	Mean (SD)
PAM		
Score 0-100	62.7 (13.1)^a^	60.9 (15.4)^a^
		
SF-36		
Physical functioning	82.4 (17.8)	81.8 (16.6)
Role functioning – physical	49.0 (43.0)	51.7 (43.2)
Role functioning – emotional	65.3 (40.1)^b^	78.5 (37.8)^b,+^
Vitality	57.9 (17.9)^b^	60.0 (16.4)^b^
Mental health	69.8 (15.8)^b^	76.5 (14.6)^b,+^
Social functioning	71.2 (20.2)	80.5 (19.8)^+^
Bodily pain	75.0 (23.6)	74.8 (21.1)
General health	57.0 (18.5)	58.8 (17.9)
		
	Median (Range)	Median (Range)
IPAQ (MET-min/week)		
Walking	396 (0-19404)	594 (0-108660)
Moderate activity	1420 (0-13220)	1560 (0-11220)
Vigorous activity	0 (0-9600)	360 (0-8160)^*^
Total activity	2793 (0-25569)	3724.2 (0-17598)

^a^N=68

^b^N=72

^*^different from baseline; *P*<.05

^+^different from baseline; *P*<.01

### Focus Group Results

Results indicated that participants (n=6) were pleased with having access to information and being able to re-read information. At the same time, focus group participants indicated that the educational materials, feedback, and advice could benefit from tailoring (ie, making the information more personal) and could be presented in a more visually attractive manner. Participants also expressed interest in being able to make and change appointments online and in obtaining access to the full EMR, and felt that providing regular updates and having their health professionals encourage them to use MijnAVL could contribute to sustained use.

### Questionnaire for Health Professionals

Twenty-four professionals, including medical oncologists, surgeons, radiotherapists, and nurse specialists (response rate 73%) completed the questionnaire. Thirty-eight percent (9/24) indicated that their patients who had access to MijnAVL asked questions about the program (eg, about the overview of appointments, login procedures, access to the EMR). Twenty-one percent (5/24) indicated that they received questions about the content of the EMR (eg, requests for explanation of jargon, interpretation of reports). Thirty-three percent (8/24) reviewed their patients’ PRO results and some discussed those results with their patients. One-quarter of the health professionals who had completed the questionnaire believed that their patients having had access to MijnAVL led to an increased workload (ranging from several to more than ten minutes).

## Discussion

The results of this study support the feasibility of MijnAVL, an interactive portal for breast cancer survivors. Use varied widely between participants, with highest levels of use being observed for those on-treatment. The scores on the UTAUT components and the WUS indicated that satisfaction with MijnAVL was relatively high, with overview of appointments and access to the EMR being most highly rated. Although both the questionnaire and focus group results suggested that participants perceived having more knowledge and control over their disease due to exposure to MijnAVL, the PAM scores did not reflect such change over time. Participants’ scores on three quality of life domains and their level of vigorous activity improved significantly from pre- to post-intervention. The focus group yielded useful feedback for improving MijnAVL (particularly the need to further tailor the information provided). Health professionals indicated that although some patients asked questions about MijnAVL, it led to only a modest increase in workload.

Ratings of experiences with MijnAVL were somewhat lower than expectations beforehand. The focus group confirmed that MijnAVL did not fully live up to the expectations, primarily because information and advice were not sufficiently tailored to individual needs. Two literature reviews have also indicated that tailored information matching user needs is an important feature of successful eHealth interventions [23,24]. Nevertheless, participants expressed high levels of satisfaction with MijnAVL and used it quite regularly, particularly individuals on-treatment. This result probably reflects the fact that during treatment, more relevant information was available, including updates of the patients’ EMR, thus leading to more logins [25].

Because use of MijnAVL was on a voluntary basis, it was not possible to calculate adherence rates. However, the dropout rate was relatively low compared to other eHealth interventions [26] and the completion rate of PROs was relatively high. This result may reflect the fact that MijnAVL is directly linked to the EMR, and thus may be perceived by patients as being an integral part of the health care process; something that has been found to be an important characteristic of successful eHealth applications [24]. Another factor that might have contributed to the sustained use of MijnAVL was the use of automatic email reminders for the completion of questionnaires [27]. To further improve the use of MijnAVL it might be beneficial not only to provide more tailored information and advice, but also to facilitate social support, to have health professionals encourage their patients to use the system, and to provide feedback (eg, regarding PRO data) [23,25].

Linkage to the EMR is an important feature of MijnAVL. Importantly, clinical staff supported sharing test results and other EMR information, with the exception of their personal notes. Post-intervention, approximately 40% (10/24) of professionals indicated that participants had posed questions about MijnAVL, half of which were about medical topics. Twenty-five percent (8/24) of the health professionals indicated some increased workload due to their patients using MijnAVL. This is in contrast with an earlier study that indicated no increased workload resulting from patients’ access to lab test results [28]. This difference could be due to the fact that MijnAVL provided access to a wider range of EMR data. Additional studies are needed to better understand the impact of portals such as MijnAVL on the content and processes of care. It would be particularly useful to conduct observational studies that document the actual (versus self-reported) influence of portals on doctor-patient communication.

Despite predominantly positive experiences with MijnAVL, the impact we measured on patient empowerment, quality of life, and physical activity were relatively modest. Although participants indicated that they felt better informed because of their exposure to MijnAVL, this did not translate into increased levels of patient empowerment, at least as measured by the PAM. It is possible that the PAM is not sufficiently sensitive to changes in empowerment over time, and that more specific questions are needed to detect such changes. It may also be the case that only a relatively brief exposure to a patient portal, as was the case in the current study, may be insufficient to facilitate increased feelings of empowerment. Similarly, the modest observed improvement in quality of life and physical activity over time may be due to the relatively short exposure to MijnAVL, as well as to the absence of sufficiently tailored information. The impact of MijnAVL may be enhanced by combining its interactive features with face-to-face contacts that may increase motivation and adherence [13].

In the next version of MijnAVL we intend to incorporate more tailored information and advice based on individual information needs. Such needs may vary as a function of age, sex, educational level, ethnicity, and stage of disease [29]. We also intend to link PRO data with clinical pathways and treatment guidelines, using a stepped care approach, starting with self-management options, followed by referral to more formal forms of intervention and care, where appropriate. Finally, the physical activity support program could be improved by taking greater account of personal (eg, preferred type of exercise) and environmental (eg, support, resources) factors when providing advice. It may be particularly important to attend to the needs of those who use the portal during the period of active treatment, as we observed a decrease in the amount of physical activity in half of this group.

Our study has several limitations that need to be considered. First, all participants were experienced Internet-users and willing to use MijnAVL. This consistency may, to some degree, overestimate the feasibility and accessibility of the program, limiting generalizability to the larger population of cancer survivors. In future studies it would be helpful to also include individuals who are less experienced Internet-users or who are less prepared to use an eHealth application, and compare their needs and experiences. Second, the absence of a control group and of information about participants’ use of other (educational) tools or mobile apps does not allow us to attribute the observed efficacy to the use of MijnAVL. Third, the data on physical activity were based on self-reports, which are known to be less reliable than objective measures such as accelerometers [30]. Despite these limitations, our study nevertheless is one of the first to investigate the feasibility of an interactive portal for breast cancer survivors that is fully integrated into the care trajectory and the hospital information system. In addition, the objective log data provided reliable information on the actual use of the portal.

**Table 5:** Outcome measures for participants on-treatment and off-treatment separately.

	On-treatment (N=38)		Off-treatment (N=35)	
	Baseline	Post-intervention	Baseline	Post-intervention
	Mean (SD)	Mean (SD)	Mean (SD)	Mean (SD)
PAM				
Score 0-100	62.7 (13.2)^a^	61.5 (16.8)^a^	62.7 (13.2)^b^	60.1 (13.7)^b^
				
SF-36				
Physical functioning	82.2 (20.8)	80.4 (18.3)	82.5 (14.1)	83.4 (14.6)
Role functioning – physical	45.4 (42.3)	41.4 (42.4)	52.9 (46.1)	62.9 (41.7)
Role functioning – emotional	58.6 (42.6)^a^	77.0 (38.0)^a,*^	72.4 (36.6)	80.0 (38.1)
Vitality	57.4 (18.2)^a^	59.1 (17.0)^a^	58.4 (17.8)	61.0 (16.0)
Mental health	66.3 (14.9)^a^	77.4 (14.5)^a,+^	73.6 (16.1)	75.4 (14.8)
Social functioning	69.4 (21.7)	78.0 (21.7)	73.2 (18.5)	83.2 (17.4)^+^
Bodily pain	71.3 (27.6)	74.6 (18.2)	79.1 (17.8)	75.0 (24.1)
General health	55.0 (21.1)	55.9 (19.4)	59.1 (15.3)	62.0 (15.8)
				
	Median (Range)	Median (Range)	Median (Range)	Median (Range)
IPAQ (MET-min/week)				
Walking	330.0 (0-19404)	651.8 (0-9890)	627.0 (0-3762)^c^	462.0 (0-10866)^c^
Moderate activity	832.5 (0-13220)	1039.5 (0-8310)	1835.0 (0-6800)^c^	2430.0 (0-11220)^c^
Vigorous activity	0.0 (0-9600)	360.0 (0-8160)	0.0 (0-4920)^c^	405.0 (0-7080)^c^
Total activity	1921.5 (0-25569)	2542.5 (0-13374)	3326.0 (0-13106)^c^	4005.0 (0-17598)^c^

^a^N=37

^b^N=31

^c^N=33

^*^different from baseline; *P*<.05

^+^different from baseline; *P*<.01

### Conclusion

Our findings suggest that MijnAVL is a feasible eHealth application for breast cancer survivors. MijnAVL could be further improved by including more visually attractive and tailored information, and adapted to individual information needs where possible. Research with a controlled design, a longer follow-up period, and including more specific outcome measures is needed to further document the effects of such an interactive portal.

## Abbreviations

AVL: Antoni van Leeuwenhoek hospital
eHEALS: eHealth literacy scale
EMR: Electronic Medical Record
IPAQ: International Physical Activity Questionnaire
MET: Metabolic Equivalent of Task
PAM: Patient Activation Measure
PRO: Patient-reported outcome
SF-36: Short-Form 36-Item Health Survey
UTAUT: Unified Theory of the Acceptance and Use of Technology
WUS: website user satisfaction questionnaire
